# Diagnostic gene signatures and aberrant pathway activation based on m6A methylation regulators in rheumatoid arthritis

**DOI:** 10.3389/fimmu.2022.1041284

**Published:** 2022-12-13

**Authors:** Qishun Geng, Xiaoxue Cao, Danping Fan, Xiaofeng Gu, Qian Zhang, Mengxiao Zhang, Zheng Wang, Tingting Deng, Cheng Xiao

**Affiliations:** ^1^ China-Japan Friendship Hospital (Institute of Clinical Medical Sciences), Chinese Academy of Medical Sciences and Peking Union Medical College, Beijing, China; ^2^ Institute of Clinical Medical Sciences, China-Japan Friendship Hospital, Beijing, China; ^3^ Beijing Key Laboratory of Research of Chinese Medicine on Prevention and Treatment for Major Diseases, Experimental Research Center, China Academy of Chinese Medical Sciences, Beijing, China; ^4^ Biotechnology Research Institute, Chinese Academy of Agricultural Sciences, Beijing, China; ^5^ Laboratory for Bone and Joint Diseases, RIKEN Center for Integrative Medical Sciences, Tokyo, Japan; ^6^ Department of Emergency, China-Japan Friendship Hospital, Beijing, China

**Keywords:** rheumatoid arthritis, N6-methyladenosine, IGF2BP3, cell cycle, M1 macrophages

## Abstract

**Purpose:**

Rheumatoid arthritis (RA) is a chronic autoimmune disease (AD) characterized by persistent synovial inflammation, bone erosion and progressive joint destruction. This research aimed to elucidate the potential roles and molecular mechanisms of N6-methyladenosine (m6A) methylation regulators in RA.

**Methods:**

An array of tissues from 233 RA and 126 control samples was profiled and integrated for mRNA expression analysis. Following quality control and normalization, the cohort was split into training and validation sets. Five distinct machine learning feature selection methods were applied to the training set and validated in validation sets.

**Results:**

Among the six models, the LASSO_λ-1se model not only performed better in the validation sets but also exhibited more stringent performance. Two m6A methylation regulators were identified as significant biomarkers by consensus feature selection from all four methods. IGF2BP3 and YTHDC2, which are differentially expressed in patients with RA and controls, were used to predict RA diagnosis with high accuracy. In addition, IGF2BP3 showed higher importance, which can regulate the G2/M transition to promote RA-FLS proliferation and affect M1 macrophage polarization.

**Conclusion:**

This consensus of multiple machine learning approaches identified two m6A methylation regulators that could distinguish patients with RA from controls. These m6A methylation regulators and their target genes may provide insight into RA pathogenesis and reveal novel disease regulators and putative drug targets.

## Introduction

Rheumatoid arthritis (RA) is a chronic autoimmune disease (AD) characterized by tumour-like hyperplasia of synovial tissue, persistent synovial inflammation, bone erosion and progressive joint destruction (1). RA usually occurs in middle-aged women. Currently, we attribute the development of RA to genetic and environmental factors, such as smoking, obesity, stress, neurodepression, and female hormones. Patients with RA have a higher risk of developing malignancies than the general population (2). Recently, the management of clinical symptoms and complications in RA patients has received increasing attention from medical workers (3, 4). An in-depth understanding of the mechanisms underlying RA occurrence and development can help to detect RA and its complications earlier so that measures can be taken to control the development and reduce the activity of the disease.

Previous studies have shown that T/B lymphocytes, macrophages, fibroblast-like synoviocytes (FLSs) and other cells are involved in the pathogenesis of RA (5). Activated FLSs in synovial tissue exacerbate the inflammatory response by secreting proinflammatory factors, chemokines and cell adhesion molecules, which can recruit additional immune cells to synovial tissue (6). Although the pathogenesis of RA remains incompletely elucidated, immune cells and FLSs undoubtedly play a crucial role in the progressive joint destruction and inflammatory response (7). Therefore, studying strategies to inhibit the proliferation and migration of FLSs and the inflammatory response in RA is highly important for elucidating the disease mechanism and developing treatments.

The study of epigenetics, especially RNA modifications, is a hotspot in life science research. Recently, with the development of the first RNA N6-methyladenosine (m6A) map by Cornell University and the discovery of its ubiquity in mRNA, transcriptional modification has gradually become the focus of the biomedical community (8). Among RNA modifications, m6A accounts for the largest proportion of base modifications in mRNAs and functions to regulate RNA stability, protein synthesis and translation; stem cell stress responses, cytotoxic stress responses; and mRNA export (9, 10). Currently, the known m6A methylation regulators consist of eight writers (METTL3, METTL14, WTAP, KIAA1429, RBM15, RBM15B, CBLL1 and ZC3H13), two readers (FTO and ALKBH5) and thirteen erasers (YTHDF1, YTHDF2, YTHDF3, YTHDC1, YTHDC2, HNRNPC, HNRNPA2B1, IGF2BP1, IGF2BP2, IGF2BP3, FMR1, ELAVL1 and LRPPRC) (11). Previous studies have shown that these regulators are involved in biological processes (BPs) such as cell differentiation and apoptosis and immune regulation, which are closely related to cancers and immune diseases (12–14). However, few studies have addressed the regulatory mechanism of m6A in RA, and more attention is needed.

**Figure 1:**
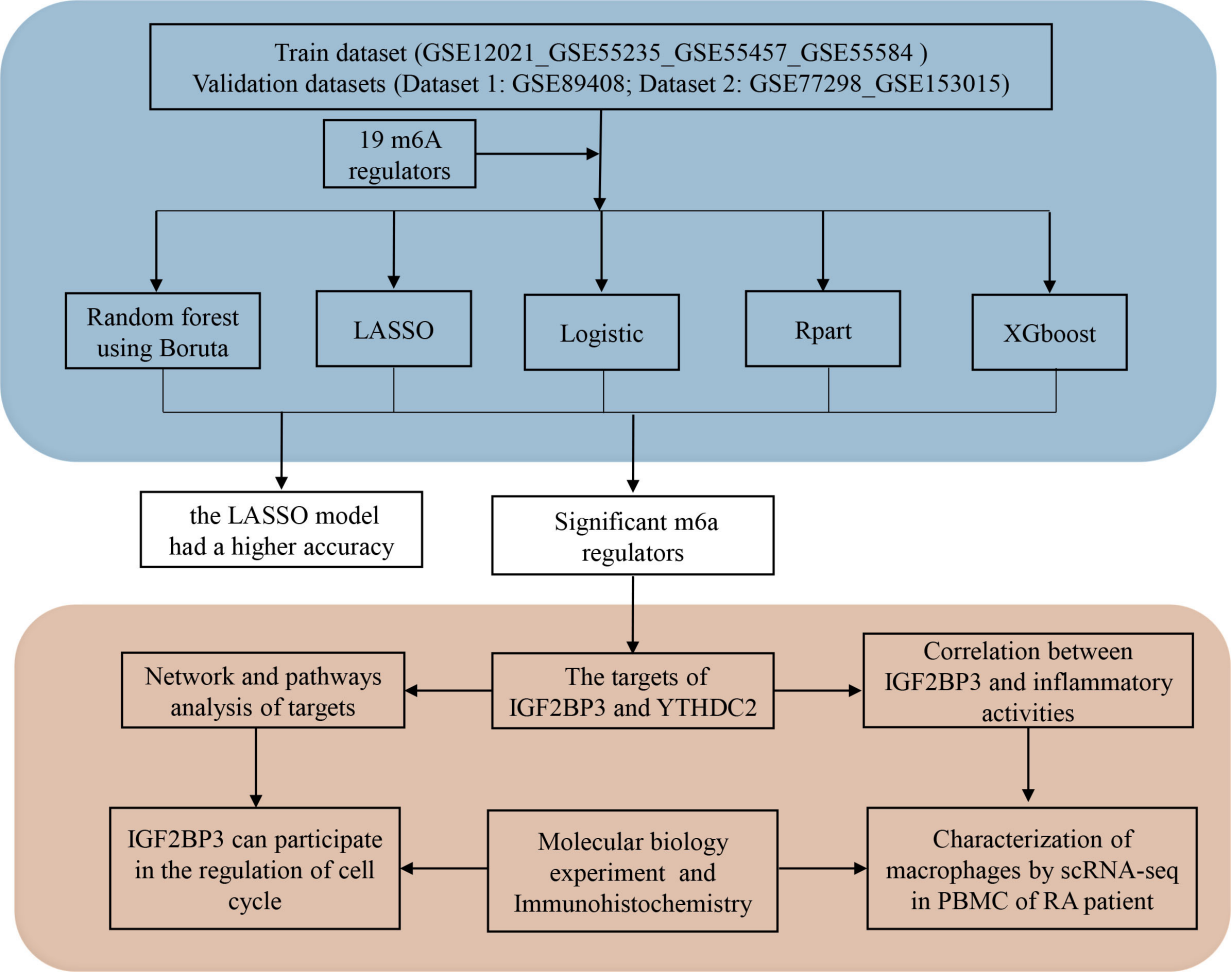
Diagram of the study.

In this study, we selected 19 m6A methylation regulators with expression data in the GSE12021, GSE55235, GSE55457, GSE55584, GSE77298 and GSE153105 datasets. Based on five distinct supervised machine learning approaches, we assessed the potential of these m6A methylation regulators as diagnostic tools by creating binary predictive classification models and assessing their accuracy. Then, by analysing the target genes and pathways of the m6A methylation regulators, we gained a further understanding of the roles of m6A methylation regulators in the pathogenesis of RA (**Figure 1**). This study is of great significance for elucidating the potential roles and molecular mechanisms of m6A methylation regulators in RA and for exploring new RA biomarkers.

## Materials and methods

### Dataset collection and processing

Data for 384 samples were accessed *via* the Gene Expression Omnibus (GEO) repository (**Supplementary Table 1**). The data from GSE12021, GSE55235, GSE55457 and GSE55584 were retrieved from the Affymetrix^®^ GPL96 platform (Human Genome U133A Array), and the data from GSE77298 and GSE153105 were retrieved from the Affymetrix^®^ GPL570 platform (Human Genome U133 Plus 2.0 Array). The raw data from the Affymetrix^®^ platforms were processed *via* the robust multiarray averaging (RMA) algorithm implemented in the Affy package. After removal of batch effects with the ComBat algorithm, the training dataset was generated by combining the GEO datasets from the Affymetrix^®^ GPL96 platform. Validation dataset 1 was generated by combining the GEO datasets from the Affymetrix^®^ GPL570 platform. GSE89408 (platform: GPL1154) was considered validation dataset 2. In this research, for comparison with the RA group, we defined healthy individuals and patients with osteoarthritis (OA) as the control group.

The samples in GSE12021, GSE55235, GSE55457, GSE55584, GSE77298 and GSE153105 were extracted from synovial tissues. The samples in GSE90081 were taken from peripheral blood mononuclear cells (PBMCs). To investigate the relationship between IGF2BP3 expression and M1 macrophages, single-cell RNA sequencing (scRNA-seq) data from the GSE159117 dataset were analysed.

### Cell lines and cell transfection

RA-FLSs were isolated from RA synovium. The cells were maintained in Dulbecco’s modified Eagle’s medium (DMEM) (Gibco, Grand Island, NY, USA) supplemented with 15% foetal bovine serum (FBS) (Thermo, USA) and cultured at 37°C in 5% CO_2_ and saturated humidity. The ethics committee of China-Japan Friendship Hospital approved the research (approval number 2021-153-K111).

To silence the expression of IGF2BP3, an IGF2BP3 siRNA (siIGF2BP3) and a control siRNA (siNC) were chemically synthesized by Tsingke Biotechnology Co., Ltd (Beijing, China) and transfected into RA-FLSs and RAW 264.7 cells. The siIGF2BP3 target sequences are shown below: human si-IGF2BP3, 5’- GCAAAGGATT CGGAAACTT -3’; mouse si-Igf2bp3, 5’- GGAGGUGCUGGAUAGUUUACU -3’. JetPRIME^®^ Transfection Reagent was used for cell transfection (Polyplus Transfection, USA).

### Random forest optimization using boruta

Boruta has high feature variable selection accuracy in biological data. We used the default settings in the Boruta package (v7.0.0) to evaluate variable importance with 300 iterations (15). After 300 iterations, the confirmed variables were identified. Then, these confirmed variables selected by Boruta were used to construct a random forest model by using the caret package (v.6.0-92). After tuning and modelling, the final selected model was obtained and used to determine whether the subjects were RA patients or non-RA patients.

### Regression partition tree

Rpart is a commonly used decision tree modelling method with a good visualization effect and straightforward results. We used the Rpart (v4.1-15) package to build a classification tree model. To avoid overfitting, some rules with weak classification and descriptive abilities were removed to improve the prediction accuracy. The classification tree model was optimized based on the minimum Xerror value, and the optimal classification tree model was used to determine whether the subjects were RA patients or non-RA patients.

### Least absolute shrinkage and selection operator

LASSO has the advantage of preserving subset shrinkage and is a biased estimator for dealing with data with complex collinearity. Lasso allows a more refined model to be obtained by constructing a penalty function such that some coefficients are compressed and some coefficients are set to zero (16). LASSO-penalized logistic regression was performed with the glmnet package (version 4.1-4), which then calculated two automatic λ values—one that minimizes the binomial deviance and one representing the largest λ that is still within 1 standard error of the minimum binomial deviance. Both λ values (λ-min=0.02395, λ-1se=0.09203) were selected and used to refit the model, which resulted in a stricter penalty that allowed us to reduce the number of covariates even further than with the former λ. A probability threshold of > 0.5 was used to determine whether the subjects were RA patients or non-RA patients.

### Extreme gradient boosting

XGBoost is an extreme gradient boosting algorithm that ranks features from most important to least important and has been used very effectively in diverse classification problems. Based on the default parameters, we used the XGBoost package (version 1.6.0.1) to build the final model for disease diagnosis and rank the features by importance. Features contributing to more than a 5% improvement in accuracy to their branches were selected as ‘important’ (17). The trained model was used to determine whether the subjects were RA patients or non-RA patients.

### Logistic regression

Logistic regression is a machine learning method used to solve binary classification problems to estimate the likelihood of an event. The glmnet package (version 4.1-4) was used to build the final model for disease diagnosis, which was used to determine whether the subjects were RA patients or non-RA patients.

### Pathway analysis

M6A2Target (http://m6a2target.canceromics.org/) is a comprehensive database for determining the target genes of writers, erasers and readers (WERs) of m6A modification. It integrates highly confidential targets validated by low-throughput experiments and potential targets with binding evidence indicated by high-throughput sequencing or inferred from m6A WER perturbation followed by high-throughput sequencing. The gene targets of the more important m6A regulators in disease diagnosis were inferred using m6A2Target (18). Then, ClueGO (version 3.0.3) was used for BP functional annotation analysis of the gene targets (19). The clusterProfiler package (version 4.2.2), a universal enrichment tool for interpreting omics data, was used for functional enrichment analysis.

### scRNA-seq analysis

First, we imported the H5 file and converted the data to a Seurat object. Then, with the Seurat (version 4.1.1) package, data quality control and clustering were performed on the PBMC population. Each cell subset was annotated based on the celldex package (version 1.4.0).

### Real-time qPCR analysis and western blot analysis

RNA isolation and RT–qPCR analysis were carried out according to previous studies (20). β-actin served as an internal control. The sequences of the primers used in the experiment are as follows. Human IGF2BP3: 5′- TCGAGGCGCTTTCAGGTAAA-3′ (forward), 5′- AAACTATCCAGCACCTCCCAC-3′ (reverse). Mouse Igf2bp3: 5′- CCTGGTGAAGACGGGCTAC-3′ (forward), 5′- TCAACTTCCATCGGTTTCCCA-3′ (reverse).

Protein extraction and Western blot analysis were carried out according to previous studies (20). The primary antibodies included rabbit anti-IGF2BP3 (1:1000, Proteintech, Chicago, USA), anti-CCNB1 (1:1000, Shanghai, China) and anti-C-Myc (1:2,000, Cell Signaling Technology, Beverly, MA, USA). Band densities on autoradiograms were densitometrically quantified (Quantity One software; Bio-Rad), with GAPDH serving as the internal control.

### Cell viability assay and cell cycle analysis

The cell viability assay was performed 24 h after transfection of siNC and siIGF2BP3 with a CCK-8 kit from Beyotime (Beijing, China). After transfection, cells were plated in 96-well dishes at a concentration of 5 × 10^3^ cells/well and cultured in DMEM containing 15% FBS for cell attachment. Cell viability was measured with CCK-8 reagent following the manufacturer’s protocol at the indicated time points (24, 48 and 72 h).

Cell cycle analysis was performed 48 h after transfection of siNC and siIGF2BP3. Cells were washed twice with ice-cold PBS, harvested, and fixed with 70% ethanol at 4°C overnight. Then, the cells were stained with a Cell Cycle and Apoptosis Analysis Kit (Beyotime, Beijing, China) at 37°C for 30 minutes and detected by flow cytometry (Becton-Dickinson, San Jose, CA, USA). Cell cycle distributions were analysed with ModFit LT 3.1 software (verity Software House, Inc., Topsham, ME, USA).

### Flow cytometric analysis and enzyme linked immunosorbent assay

Analysis was performed 48 h after transfection of siNC and iIGF2BP3. After 6h of LPS (100ng/ml) stimulation, cells were collected and washed with PBS. Subsequently, the cells were directly surface stained using anti-CD86 antibodies (Biolegend, California, USA) for 20 min at 4°C. Signals were detected by flow cytometry (Becton-Dickinson, San Jose, CA, USA). Data analysis was conducted with FlowJo software version 10.0 (Tree Star, Inc., Ashland, OR, USA).

After transfection and stimulation, the cell supernatant was collected. According to the protocol of Mouse TNF-alpha ELISA Kit (ABclonal, Wuhan, China), the content of TNF-α in cell supernatant was detected.

### Immunohistochemistry

The synovium tissues of six RA patients and six OA patients are obtained from China-Japan Friendship Hospital. Sample processing and data analysis were performed as previously described (20). The ethics committee of China-Japan Friendship Hospital approved the research (approval number 2021-153-K111).

### Statistical analyses

Statistical analyses were performed using GraphPad Prism Software (GraphPad Software, San Diego, CA) and R version 4.0.4 software (Institute for Statistics and Mathematics, Vienna, Austria; https://www.r-project.org). We used a leave-one-out (LOO) cross-validation approach to evaluate the performance of the classifiers in the training set. Student’s t test was used for comparisons between groups. Measurement data are expressed as the means ± standard deviations, and *P*< 0.05 indicates statistical significance.

## Results

### Performance of RA classification approaches using the m6A regulators

**Figure 2:**
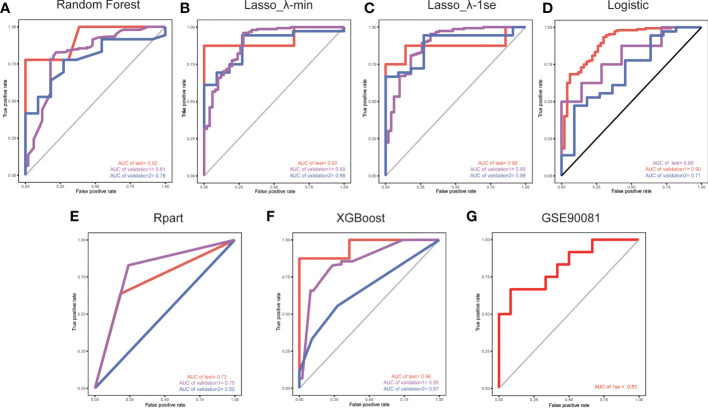
ROC curves for validation set 1 and validation set 2, with the model trained on a separate set. The red lines indicate the models trained using a LOO cross-validation approach across the training set. We used five methods to develop models based on the training set: **(A)** a random forest wrapper (Boruta), **(B)** LASSO_λ-min, **(C)** LASSO_λ-1se, **(D)** logistic regression, **(E)** regression partition trees (Rpart) and **(F)** extreme gradient boosting (XGBoost). **(G)** ROC curve of the LASSO_λ-1se model in whole blood samples.

Considering the important role of m6A methylation regulators in tumour and immune disease progression, we used a public dataset to comprehensively explore the importance of 19 m6A methylation regulators for RA diagnosis. Based on the expression levels of these 19 m6A methylation regulators, a disease diagnosis model (RA *vs*. non-RA) was constructed using five different machine learning methods: random forest optimization using Boruta, Rpart, LASSO, XGBoost and logistic regression. The cross-validation performance in the training set is presented in **Supplementary Table 2**. The accuracy and AUC of all models except for the Rpart model were greater than 0.8. To compare the performance of each machine learning method, we observed the performance of each model as a classifier in the validation sets. The performance of each machine learning method in the validation sets was also variable (**Tables 1**, **2**; **Figures 2A-F**). In validation dataset 1, the logistic regression model and LASSO_λ-min model had the highest AUC (0.90), but the LASSO_λ-min model had a higher accuracy (0.901). The Rpart model had the lowest AUC (0.8). In validation dataset 2, the LASSO_λ-min model and LASSO_λ-1se model had the highest accuracy (0.89) and AUC (0.88). Among the models, the Rpart model had the poorest performance. In addition, the number of m6A methylation regulators selected by each machine learning method differed, with Boruta selecting the most (14 regulators) and the Rpart model selecting just one regulator. Considering the performance of each machine learning method in the validation sets and the number of regulators that it selects in the models, the LASSO_λ-1se model not only performed better in the validation sets but also exhibited more stringent in variable screening. These results indicate that the LASSO_λ-1se model has good clinical application value and practicality. Therefore, we further compared the performance of the LASSO_λ-1se model in whole blood samples and calculated an AUC value of 0.83 (**Figure 2G**), further suggesting that the LASSO_λ-1se model has clinical application prospects in blood-based diagnosis of RA.

**Table 1:** Model performance of the six classifiers in validation set 1: A random forest wrapper (Boruta), LASSO_λ-min, LASSO_λ-1se, logistic regression, regression partition trees (Rpart) and extreme gradient boosting (XGBoost).

	Random forest	LASSO_min	LASSO_1se	Logistic	Rpart	XGBoost
**Regulators selected by model, n**	14	11	4	13	1	5
**Best threshold**	0.481 (0.22,0.829)	0.520 (0.28,0.961)	1.280 (0.26,0.895)	-293.891 (0.3,0.934)	0.5014 (0.24,0.829)	0.903 (0.22,0.809)
**Sensitivity**	0.78	0.72	0.74	0.7	0.76	0.78
**Specificity**	0.8289	0.961	0.8947	0.9342	0.8289	0.8092
**Positive predictive value**	0.6	0.8571	0.6981	0.7778	0.5938	0.5735
**Negative predictive value**	0.9197	0.9125	0.9128	0.9045	0.913	0.9179
**Acuracy (95%)**	0.8168 (0.7565~0.8676)	0.901 (0.8512-0.9385)	0.8564 (0.8004-0.9017)	0.8762 (0.8227-0.9183)	0.812 (0.7511-0.8633)	0.802 (0.7403-0.8546)
**AUC (95%)**	0.811 (0.735-0.888)	0.895 (0.841-0.948)	0.89 (0.830-0.944)	0.899 (0.847-0.95)	0.794 (0.728-0.861)	0.853 (0.792-0.914)

**Table 2:** Model performance of the six classifiers in validation set 2: A random forest wrapper (Boruta), LASSO_λ-min, LASSO_λ-1se, logistic regression, regression partition trees (Rpart) and extreme gradient boosting (XGBoost).

	Random forest	LASSO_min	LASSO_1se	Logistic	Rpart	XGBoost
**Regulators selected by model, n**	14	11	4	13	1	5
**Best threshold**	0.693 (0.273,0.778)	-2.229 (0.273,0.944)	-1.588 (0.273,0.944)	-7331.730 (0.091,0.472)	NA	0.007 (0.273,0.556)
**Sensitivity**	0.7273	0.7273	0.7273	0.9091	1	0.7273
**Specificity**	0.7778	0.9444	0.9444	0.4722	0	0.5556
**Positive predictive value**	0.5	0.8	0.8	0.3448	0.234	0.3333
**Negative predictive value**	0.9032	0.9189	0.9189	0.9444	NA	0.8696
**Acuracy (95%)**	0.766 (0.6197, 0.877)	0.8936 (0.769,0.9645)	0.8936 (0.769,0.9645)	0.5745 (0.4218-0.7174)	0.234 (0.123-0.3803)	0.5957 (0.4427-0.7363)
**AUC (95%)**	0.782 (0.641-0.923)	0.884 (0.780-0.988)	0.881 (0.778-0.984)	0.707 (0.525-0.889)	0.5	0.667 (0.518-0.816)

NA, Not Applicable.

### The more important m6A methylation regulators in the RA classification

**Figure 3:**
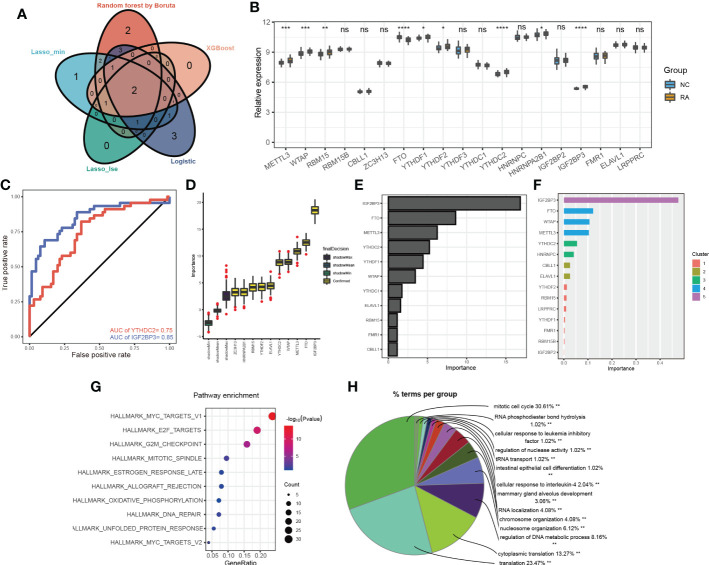
The more important m6A methylation regulators in RA classification. **(A)** Venn diagram of the m6A methylation regulators selected by the different machine learning methods; **(B)** the expression levels of 19 m6A methylation regulators in the training dataset; **(C)** the ROC curves for IGF2BP3 and YTHDC2 in the training set; the importance of the 19 m6a methylation regulators calculated by the Boruta **(D)**, Rpart **(E)** and XGBoost **(F)** algorithms; KEGG pathway **(G)** and BP **(H)** enrichment analyses of the gene targets of IGF2BP3 and YTHDC2 *p < 0.05, **p < 0.01, ***p < 0.001, ****p < 0.0001, ns (p > 0.05).

Different candidate biomarkers were selected by these multivariable machine learning methods. However, biomarkers often have equal accuracy and importance (17). Considering the poorest performance of the Rpart model, we focused on the overlapping m6A methylation regulators selected by the different machine learning methods, including of random forest optimization using Boruta, LASSO, XGBoost and logistic regression (**Figure 3A**; **Supplementary Table 3**). Two of the overlapping m6A methylation regulators were selected by every model: IGF2BP3 and YTHDC2. The expression levels of the 19 m6A methylation regulators were further compared in the training dataset. The expression levels of IGF2BP3 and YTHDC2 were significantly different in RA and non-RA patients (**Figure 3B**). More importantly, based on transcript levels, IGF2BP3 and YTHDC2 also performed well in the diagnosis of RA in the training set (**Figure 3C**), with AUC values of 0.85 and 0.75, respectively. In addition, when the Boruta (**Figure 3D**), Rpart (**Figure 3E**) and XGBoost (**Figure 3F**) algorithms were used to calculate the importance of the 19 m6A methylation regulators, IGF2BP3 and YTHDC2 were ranked high; and IGF2BP3 has the highest importance.

### Pathway and network analysis of the IGF2BP3 and YTHDC2 targets

**Figure 4:**
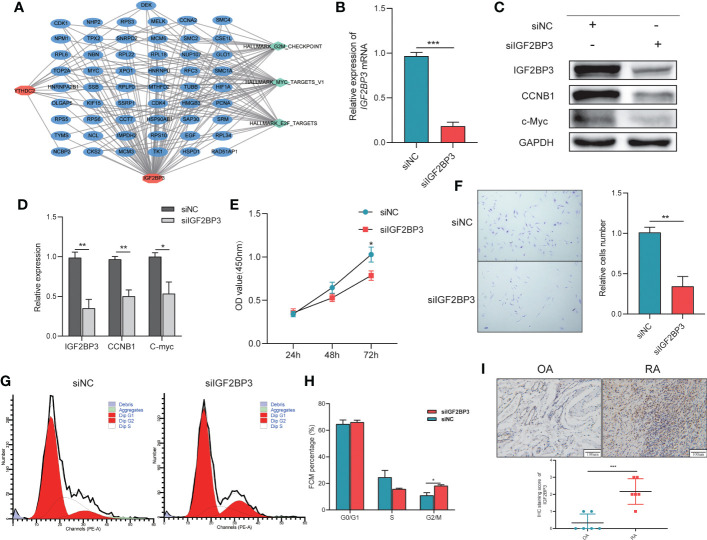
The importance of IGF2BP3 in the Viability and Cell Cycle of RA-FLSs. **(A)** The network connecting IGF2BP3 and YTHDC2 pathways and other targets; **(B)** RT−qPCR results showing the efficient depletion of IGF2BP3 expression in RA-FLSs compared with siNC-transfected RA-FLSs; **(C, D)** Expression of IGF2BP3, c-MYC and CCNB1 in RA-FLSs after transfection; **(E)** The proliferative ability of RA-FLSs after transfection was evaluated by a CCK-8 assay; **(F)** Representative images (left) and histograms (right) showing the effect of siFN1 on the cell proliferation of RA-FLSs; **(G, H)** Flow cytometric analysis was used to evaluate the cell cycle distribution of RA-FLSs after transfection; **(I)** Representative IHC staining and IHC staining score of Synovial tissues. *p < 0.05, **p < 0.01, ***p < 0.001.

To investigate the novel roles that these m6A methylation regulators play in RA and examine the related pathways, we predicted their target genes using m6A2Target. IGF2BP3 and YTHDC2 had 287 predicted gene targets in total (**Supplementary Table 4**); IGF2BP3 had 16 verified targets and 190 predicted targets, and YTHDC2 had 9 verified targets and 77 predicted targets. Based on the predicted gene targets, KEGG pathway enrichment analysis was performed using the ClusterProfiler package (version 4.2.2) to analyse the signalling pathways in which IGF2BP3 and YTHDC2 participate. These predicted gene targets were highly enriched in the following functions and pathways: MYC_TARGETS_V1, E2F_TARGETS, G2M_CHECKPOINT, MITOTIC_SPINDLE, ESTROGEN_RESPONSE_LATE, ALLOGRAFT_REJECTION, OXIDATIVE_PHOSPHORYLATION, DNA_REPAIR, UNFOLDED_PROTEIN_RESPONSE, MYC_TARGETS_V2, and so on (**Figure 3G**). Interestingly, BP functional enrichment analysis carried out by ClueGO showed that the predicted gene targets participated mainly in processes related to the mitotic cell cycle, translation, cytoplasmic translation and regulation of DNA metabolic processes, which play key roles in the occurrence and development of RA (**Figure 3H**). To better demonstrate the relationship between IGF2BP3 and YTHDC2, their predicted gene targets and the related pathways, Cytoscape (version 3.9.0) was used to construct a network, which indicated that IGF2BP3 and YTHDC2 can regulate the G2M_CHECKPOINT, MYC_TARGETS_V1 and E2F_TARGETS pathways by acting on CDK1, CDK2, MYC and other targets (**Figure 4A**).

### The importance of IGF2BP3 in the viability and cell cycle of RA-FLSs

Based on the pathway enrichment analysis results, IGF2BP3 and YTHDC2 are closely related to the cell cycle. But, when the Boruta (**Figure 3D**), Rpart (**Figure 3E**) and XGBoost (**Figure 3F**) algorithms were used to calculate the importance of the 19 m6A methylation regulators, IGF2BP3 ranked first, while YTHDC2 ranked lower. In addition, compared with YTHDC2, IGF2BP3 performed better in the diagnosis of RA (**Figure 3C**). Therefore, we further explored the regulatory effects of IGF2BP3 on the viability and cell cycle of RA-FLSs through molecular biology experiments. To explore the effects of IGF2BP3 on RA-FLSs, siRNAs were transfected into RA-FLSs. The transfection results were confirmed by RT−qPCR and Western blotting and indicated that the siRNA had a good knockdown efficiency (**Figures 4B-D**). Then, we studied the effect of IGF2BP3 on RA-FLS viability *in vitro*. The CCK-8 cytotoxicity assay revealed that downregulation of IGF2BP3 in RA-FLSs significantly reduced cell viability compared to that of the control cells (*P* < 0.05, **Figure 4E**). The cell proliferation assay also revealed that downregulation of IGF2BP3 in RA-FLSs significantly inhibited cell proliferation compared to that of the control cells (*P* < 0.05, **Figure 4F**). In addition, the flow cytometry results showed that low expression of IGF2BP3 had an obvious effect on the G2/M transition. Compared with that in the control group, the proportion of G2/M-phase cells in the siIGF2BP3 group was significantly increased (*P* < 0.05, **Figures 4G, H**). We also measured the expression of cell cycle-related proteins, showing that siIGF2BP3 reduced CCNB1 and C-MYC expression (**Figures 4C, D**). In addition, the expression of IGF2BP3 in synovial tissues of patients with OA and RA was detected. We found that IGF2BP3 expression was significantly higher in synovial tissues of RA patients, further affirming the importance of IGF2BP3 in the progression of RA (**Figure 4I**).

### Correlation between IGF2BP3 expression and inflammatory activity

**Figure 5:**
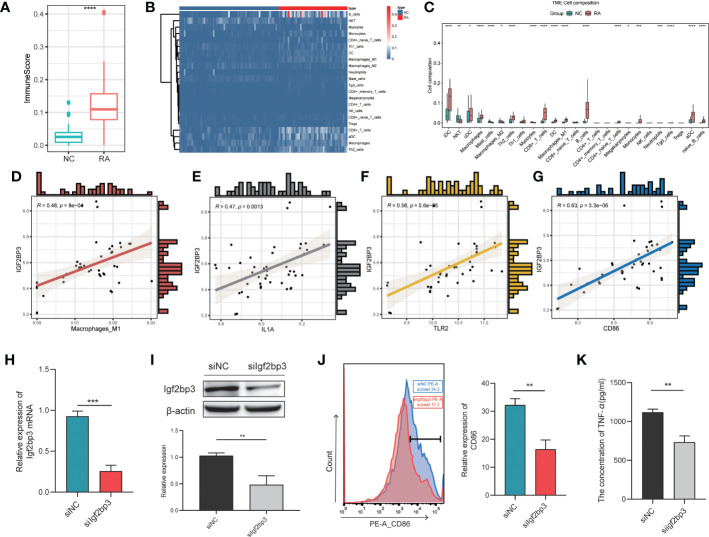
Correlation between IGF2BP3 expression and inflammatory activity. Immune scores **(A)** and proportions of immune cells **(B, C)** in the RA and NC groups; **(D)** correlation between the proportion of M1 macrophages and the expression level of IGF2BP3 in RA patients; **(E–G)** correlations between the expression levels of M1 macrophage markers (IL1A, CD86 and TLR2) and IGF2BP3; **(H, I)** RT−qPCR and Western blot analysis confirmed the efficiency of gene silencing; **(J)** expression level of CD86 in RAW264.7 cells after transfection; **(K)** the content of TNF-α in RAW264.7 cells after transfection. *p < 0.05, **p < 0.01, ***p < 0.001, ****p < 0.0001.

Increasing evidence suggests that m6A modification is an important regulator of immune response regulatory mechanisms and inflammatory regulatory networks (21). To identify the IGF2BP3-associated immune signature in RA, we determined the immune scores and the proportions of immune cells with xCell (22). First, we found significant differences in the immune score between the two groups, with higher immune scores in the RA patient group than in the NC patient group (*P* < 0.001; **Figure 5A**). Then, the proportions of immune cells were compared between the two groups. There were significant differences in the proportions of many immune cells, including interdigitating cells (IDCs), natural killer T (NKT) cells, classical dendritic cells (cDCs), macrophages, mast cells, M2 macrophages, Th2 cells, M1 macrophages, and myocytes (**Figures 5B, C**). Among these cell types, we focused on M1 macrophages because of the closely relationship between M1 macrophages and RA (23). The proportion of M1 macrophages in RA patients was significantly higher than that in control patients. In addition, we investigated the relationship between the proportion of M1 macrophages and the expression level of IGF2BP3 in RA patients and found that they were strongly correlated (**Figure 5D**). IGF2BP3 expression was also significantly correlated with the expression of M1 macrophage markers, including IL1A, CD86 and TLR2 (**Figures 5E-G**). Therefore, we thought that IGF2BP3 can participate in the regulation of M1 macrophage polarization.

To further explore the effect of IGF2BP3 on M1 macrophage polarization, we transfected RAW264.7 cells with Igf2bp3-siRNA or NC-siRNA (negative control). RT−qPCR and Western blot analysis were performed to confirm the efficiency of gene silencing and indicated that the siRNA had a good knockdown efficiency (**Figures 5H, I**). Forty-eight hours after transfection, RAW264.7 cells were treated with 100 ng/ml LPS for 24 h. Then, by measuring the expression of the surface marker (CD86) of M1 macrophages by flow cytometry, we found that the expression level of CD86 in siIgf2bp3 cells was significantly lower than that in siNC cells (**Figure 5J**). In addition, we further detected the content of TNF-a in the cell supernatant, which indicated that the content of TNF-a in siIgf2bp3 cells was lower than that in siNC cells (**Figure 5K**). These results further validated the involvement of IGF2BP3 in the regulation of M1 macrophage polarization.

### scRNA-seq revealed the relationship between IGF2BP3 expression and M1 macrophage polarization

**Figure 6:**
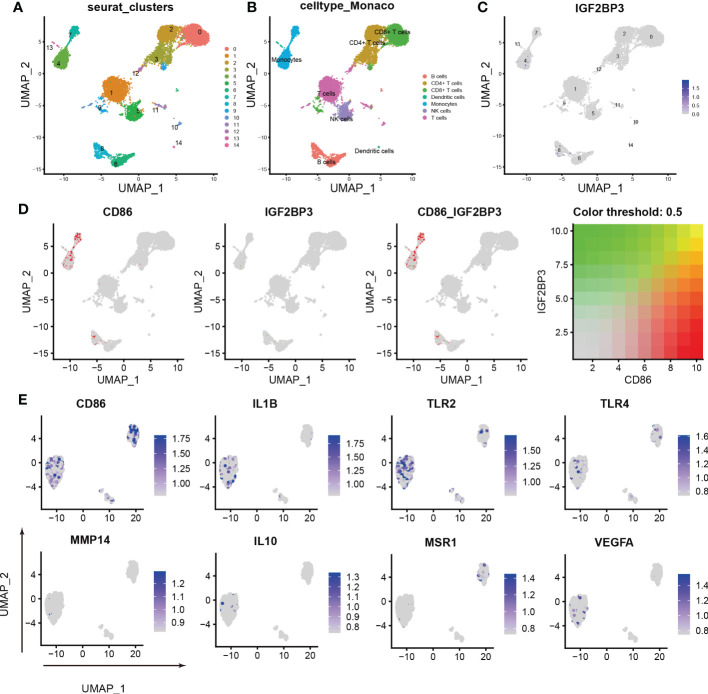
Characterization of macrophages by scRNA-seq in PBMCs. **(A)** UMAP plot showing the sources of the collected scRNA-seq cell samples; **(B)** UMAP plot showing 14 cell clusters of 7 cell types in the collected samples; **(C)** UMAP plot showing the IGF2BP3 expression level in the 14 cell clusters; **(D)** scRNA-seq analysis revealed the correlation between IGF2BP3 and CD86 expression; **(E)** UMAP plot showing the expression levels of M1 and M2 macrophage markers.

To further characterize the relationship between IGF2BP3 expression and M1 macrophage polarization, we conducted scRNA-seq in the GSE159117 dataset. Fourteen cell clusters were obtained by a combined uniform manifold approximation and projection (UMAP) analysis (**Figure 6A**). SingleR (version 1.8.1) was used to identify 7 cell types: B cells, CD4^+^ T cells, CD8^+^ T cells, dendritic cells, monocytes, NK cells and T cells (**Figure 6B**). IGF2BP3 was found to be expressed mainly on monocytes and B cells among the seven cell types (**Figure 6C**; clusters 4 and 8). Macrophages are the main type of cell derived from monocytes. Therefore, the relationship between CD86 and IGF2BP3 expression was explored in monocytes, and CD86 and IGF2BP3 were found to have a coexpression trend (**Figure 6D**). Then, we preliminarily investigated the expression of several macrophage markers in monocytes. M1 macrophage markers (including CD86, IL1B, TLR2 and TLR4) were significantly upregulated but M2 macrophage markers (including MSR1, IL10, MMP14 and VEGFA) were downregulated in monocytes (**Figure 6E**).

## Discussion

RA is a systemic autoimmune disorder affecting the synovium of peripheral joints. The average life expectancy of patients with RA is shorter than that of the overall population, and patients with active disease are also prone to develop various diseases, such as cardiovascular disease, pulmonary interstitial disease, and osteoporosis (24, 25). m6A methylation has been shown to be associated with tumours, neurological disorders, metabolic diseases, ADs, viral infections and so on (26). Mutations in the genes encoding m6A methylation regulators are closely associated with inflammation-related diseases, and changes in their expression levels have been observed in RA (21, 27). Therefore, exploring the diagnostic value and mechanism of m6A methylation regulators in RA is highly important for the effective treatment of RA and the improvement of its prognosis.

In this study, based on m6A methylation regulator expression profiles and consensus machine learning approaches, we constructed binary predictive classification models and assessed their accuracy. Among the models, the LASSO_λ-1se model not only performed better in the validation sets but also exhibited more stringent performance. In addition, the LASSO_λ-1se model exhibited better performance in whole blood samples, further suggesting that the LASSO_λ-1se model has application prospects in blood-based diagnosis of RA. Our primary aim in this study was to investigate the relationships between m6A methylation regulators and clinical classification rather than to develop a diagnostic tool. Combined with the comprehensive imaging, haematological and gene expression analyses, a diagnostic model of RA has more clinical diagnostic significance and higher accuracy. This study lays the foundation for the establishment of diagnostic tools by evaluating the accuracy of m6A methylation regulators for clinical classification and affirms the potential diagnostic value of m6A methylation regulators. A limitation of this study is the relatively small sample size used to generate and validate the m6A methylation regulators as classifiers. This may have led to overfitting of some models and thus to overestimation of effect sizes. To alleviate this issue, we validated each model’s diagnostic value in different published datasets and validated potentially interesting genes using molecular biology experiments. To develop accurate diagnostic tools, further studies based on larger retrospective and prospective clinical cohorts are warranted.

Machine learning provides an unbiased approach to predict patient status while also offering the potential to identify previously unknown interactions and identify novel biological signatures (17, 28). Our approach of investigating the biomarkers identified through multiple feature selection techniques increases confidence in the generation of reproducible biomarker panels and reduces the number of m6A methylation regulators for potential clinical investigation. The selected m6A methylation regulators (IGF2BP3 and YTHDC2) ranked highly in variable importance. Previous studies have shown that IGF2BP3 and YTHDC2 are closely related to cell proliferation and migration, cell cycle regulation, and immune and inflammatory regulation (29–31). In addition, the study by Fan et al. confirmed that IGF2BP3 not only was significantly overexpressed in RA synovial tissue but also might be a therapeutic target of thymopentin (TP) during RA treatment (32). The above literature reports provide supporting evidence that IGF2BP3 and YTHDC2, identified here as candidate biomarkers, may be associated with disease progression in RA, validating our machine learning approach to identify relevant m6A methylation regulator biomarkers. Pathway enrichment analysis showed that IGF2BP3 and YTHDC2 were involved in the regulation of MYC_TARGETS_V1, E2F_TARGETS, G2M_CHECKPOINT and other pathways, which are closely related to the cell cycle. In particular, IGF2BP3 not only was ranked highest by the Boruta, Rpart and XGBoost methods but also showed better diagnostic value in the training set. We focused on verifying the relationship between IGF2BP3 expression and the cell cycle and further confirmed that IGF2BP3 may affect the proliferation of RA-FLSs by regulating the G2/M transition. Inflammatory cells can secrete a large amount and variety of inflammatory factors and chemokines, leading to the activation of more FLSs and promoting their proliferation and migration, thereby further aggravating the inflammatory response in the disease (33). Among these immune cell types, M1 macrophages attracted our attention for the following three reasons: 1. M1 macrophages, also called classical macrophages, can produce proinflammatory cytokines and thus have potent microbicidal ability but are also prone to cause tissue destruction and exacerbate inflammatory processes that are detrimental to health (34); 2. The synovial lining of RA patients exhibits cell proliferation and a large amount of inflammatory cell infiltration in the interstitium. The degree of inflammatory infiltration determines the severity of the disease (35). 3. Among the inflammatory cells involved in RA, macrophages play a key role. These cells can polarize into different phenotypes and mediate the immune/inflammatory response as well as the repair phase when possible (23). By analysing the relationship between IGF2BP3 expression and M1 macrophage polarization in RA RNA-seq datasets and scRNA-seq datasets, we found that IGF2BP3 plays a crucial role in M1 macrophage polarization. CD86, also known as B7.2, is a T lymphocyte activation antigen with a molecular weight of 80 kD and can be expressed in dendritic cells, monocytes, T lymphocytes and B lymphocytes. Previous studies have shown that CD86 can serve as a marker to elevate the proportion of M1 macrophages (36, 37). By measuring the expression of CD86 by flow cytometry, we found that the expression level of CD86 in siIgf2bp3 RAW264.7 cells was significantly lower than that in siNC RAW264.7 cells. Yang et al. also showed that siIGF2BP3 can reduce MALAT1 expression, thereby impeding p38/mitogen-activated protein kinase phosphorylation and macrophage-mediated inflammation (38). These studies all further verified that IGF2BP3 can regulate macrophage polarization and inflammatory exacerbation during RA progression.

The RA diagnostic model established based on public databases had good performance in multiple validation sets. However, further validation of the diagnostic value of established models in larger independent cohorts is warranted before considering their clinical application. Furthermore, we used five machine learning feature selection algorithms on data from patient synovial tissue to identify two signature m6A methylation regulators in RA, and our findings may provide a new RA marker and reveal novel disease mechanisms. Moreover, this study is the first to confirm the effect of the m6A reader protein IGF2BP3 on the progression of RA and verify its biological function through bioinformatics analysis and molecular biology experiments. This study provides new ideas and strategies for the early diagnosis and targeted therapy of RA and has theoretical innovation prospects. Moreover, it provides theoretical support for the discovery of new markers and drug targets for RA.

## Data availability statement

The original contributions presented in the study are included in the article/**Supplementary Material**. Further inquiries can be directed to the corresponding authors.

## Ethics statement

The ethics committee of China-Japan Friendship Hospital approved the research (approval number 2021-153-K111). Written informed consent for participation was not required for this study in accordance with the national legislation and the institutional requirements.

## Author contributions

QG and CX designed and wrote the manuscript. The other authors participated in discussions associated with the manuscript and revised the manuscript. All authors contributed to the article and approved the submitted version.

## Supplementary material

Supplementary Table 1 Sample information from the different public datasets.

Supplementary Table 2 The leave-one-out (LOO) cross-validation performance in the training set a random forest wrapper (Boruta), LASSO_λ-min, LASSO_λ-1se, logistic regression, regression partition trees (Rpart) and extreme gradient boosting (XGBoost).

Supplementary Table 3 The selected genes in different machine learning methods.

Supplementary Table 4 The gene targets of IGF2BP3 and YTHDC2.
